# Perceived discrimination as a modifier of health, disease, and medicine: empirical data from the COVID-19 pandemic

**DOI:** 10.1038/s41398-022-02047-0

**Published:** 2022-07-15

**Authors:** Moriah E. Thomason, Cassandra L. Hendrix, Denise Werchan, Natalie H. Brito

**Affiliations:** 1 Department of Child and Adolescent Psychiatry, New York University Langone Health, New York, NY 10016 USA; 2 Department of Population Health, New York University Langone Health, New York, NY 10016 USA; 3 Neuroscience Institute, New York University Langone Health, New York, NY 10016 USA; 4 Department of Applied Psychology, New York University, New York, NY 10003 USA

**Keywords:** Neuroscience, Psychiatric disorders

## Abstract

Increasing reports of long-term symptoms following COVID-19 infection, even among mild cases, necessitate systematic investigation into the prevalence and type of lasting illness. Notably, there is limited data regarding the influence of social determinants of health, like perceived discrimination and economic stress, that may exacerbate COVID-19 health risks. Here, 1,584 recovered COVID-19 patients that experienced mild to severe forms of disease provided detailed medical and psychosocial information. Path analyses examined hypothesized associations between discrimination, illness severity, and lasting symptoms. Secondary analyses evaluated sex differences, timing of infection, and impact of prior mental health problems. Post hoc logistic regressions tested social determinants hypothesized to predict neurological, cognitive, or mood symptoms. 70.6% of patients reported presence of one or more lasting symptom after recovery. 19.4% and 25.1% of patients reported lasting mood or cognitive/memory problems. Perceived discrimination predicted increased illness severity and increased lasting symptom count, even when adjusting for sociodemographic factors and mental/physical health comorbidities. This effect was specific to stress related to discrimination, not to general stress levels. Further, patient perceptions regarding quality of medical care influenced these relationships. Finally, illness early in the pandemic is associated with more severe illness and more frequent lasting complaints. Lasting symptoms after recovery from COVID-19 are highly prevalent and neural systems are significantly impacted. Importantly, psychosocial factors (perceived discrimination and perceived SES) can exacerbate individual health risk. This study provides actionable directions for improved health outcomes by establishing that sociodemographic risk and medical care influence near and long-ranging health outcomes. All data from this study have been made publicly available.

## Introduction

Reports of long-ranging symptoms following recovery from primary SARS-CoV-2 (COVID-19) infection have spurred widespread interest in the nature, prevalence and etiology of lasting symptoms. Early, systematic study of the syndrome, referred to as Long COVID or Post-acute sequelae of COVID (Long COVID/PASC), points to specific physical and psychological symptoms that can last for months after acute COVID-19. Hospitalized and severely ill patients frequently report respiratory, gastrointestinal, pulmonary and neurological complaints months after recovery from acute infection [1]. Davis and colleagues conducted an international study of 3,762 Long COVID/PASC patients and found that fatigue, post-exertional malaise, and cognitive dysfunction comprised the most predominant symptom clusters [2]. Estiri and colleagues sought to determine whether Long COVID/PASC symptomology was applicable in patients representing the full spectrum of COVID-19 severity, including those not hospitalized. They extracted new diagnoses from Mass General Brigham electronic medical records (EMR), utilizing > 95,000 records, and then retrospectively examined which of these diagnoses had different prevalence in those that did or did not have COVID-19 in the past. They discovered that anosmia, dysgeusia, chest pain, chronic fatigue, shortness of breath and diabetes mellitus were elevated only in those with prior COVID-19 infection [3]. Review of available studies of Long COVID/PASC leads to the inevitable conclusion that the ‘debilitating second act’ (cf. Estiri) of COVID-19 infection is a significant public health concern. Developing strategies for intervention will require extension of prior work to rigorous evaluation of individual differences, such as age, physical and psychological health, and social inequities as critical determinants of Long COVID/PASC.

While initial studies reported predominately on fatigue and asnomia as the primary neurological sequalae of Long COVID/PASC, there is now evidence that cognitive and psychiatric symptoms are important components of lasting symptomology [4]. Data from the UK Biobank demonstrates reduction in global brain volume and neurocognitive decline in a sample of 401 individuals assessed behaviorally and with MRI before and after having COVID-19 infection [5]. Discovery of widespread neuroanatomical change following infection is fitting with diversity in neurobehavioral, cognitive and mood symptoms we have begun to recognize as typical of Long COVID/PASC [4]. Available evidence shows that COVID-19 pathogens can reach and infect central nervous system (CNS) cells and cause inflammation [6, 7]. Pathways by which SARS-CoV-2 may reach the CNS include the airways, circulating blood and the nasal cavity; see review by Proal and VanElzakker [8]. Neurological complications associated with COVID-19 may arise from SARS-CoV-2 CNS invasion, or may be secondary to robust immune response, indirect medical complications, or invasive therapies [9, 10]. Indeed, ex vivo studies confirm that COVID-19 infection is associated with elevated inflammatory markers, abnormal coagulation concentrations, increased cytokine expression, especially IL-6, IL-8 and TNF-alpha, endothelial dysfunction, hypercoagulable state, and imbalanced immune responses [11, 12]. Overall, it has become clear that amongst the impacted organs systems of the Long COVID/PASC syndrome, the brain is of central importance.

An important consideration in the study of lasting symptoms after recovery from COVID-19 is differential vulnerability. COVID-19 has had disproportionate impact on racial and ethnic minority groups, and it has been suggested that biomedical factors and social determinants of health underlie this difference [13–15]. Empirical studies corroborate this mechanistic account, demonstrating that adjusting for sociodemographic factors and comorbidities in patients that reach medical care nullifies racial/ethnic differences [16, 17]. Thus, in the study of Long COVID/PASC, social and economic stress are key health determinants that must be addressed. In particular, it may be important to consider subjective experiences of discrimination and economic stress as risk factors that would elevate physical and neurological symptoms persisting or emerging after recovery from primary infection. For example, discrimination, in any form (e.g., disability, sexual orientation, physical appearance, race/ethnicity, religion) may act as a barrier to healthcare, further increasing risk of negative health outcomes due to underuse of mental health services, lower trust in healthcare systems, and delayed or avoided treatment [18]. Chronic stress resulting from perceived discrimination is also associated with allostatic load [19, 20], which is defined as a dysregulation of the body’s physiological systems, including cardiovascular, neuroendocrine, metabolic, and immunologic systems that increases disease susceptibility and mortality. Despite the centrality of these factors, studies of lasting effects of COVID-19 infection have yet to rigorously address the role of prior health conditions, perceived equity, and stress, all of which are established critical determinants of health.

The present study was designed to address the prevalence, timing, and social determinants of lasting physical and neural symptoms in a large sample of patients that experienced mild to severe forms of COVID-19, months after recovery. We first provide a descriptive comparison between early and late COVID-19 cases, given the two-peak incidence of this pandemic. Primary analyses then test the hypothesis that frequency of, and stress from, discrimination contribute to COVID-19 illness severity and lingering symptoms in recovered patients, controlling for a variety of sociodemographic characteristics and mental and physical health morbidities. Secondary analyses address whether observed relationships are specific to stress associated with discrimination or reflect elevated stress more generally, and explore perceived quality of care as a potential buffer between predictors and outcomes. All analyses control for mental and physical health comorbidities. *Post hoc* analyses test whether primary effects are predictive of a general syndrome of lasting effects or if there is evidence that neural domains are specifically affected. We examined these questions using first-person, self-report data in a sample of 1,584 patients. All data have been made publicly available and curation/validation processes have been documented [21].

## Results

### Prevalence and type of lasting symptom complaints

**Fig. 1:**
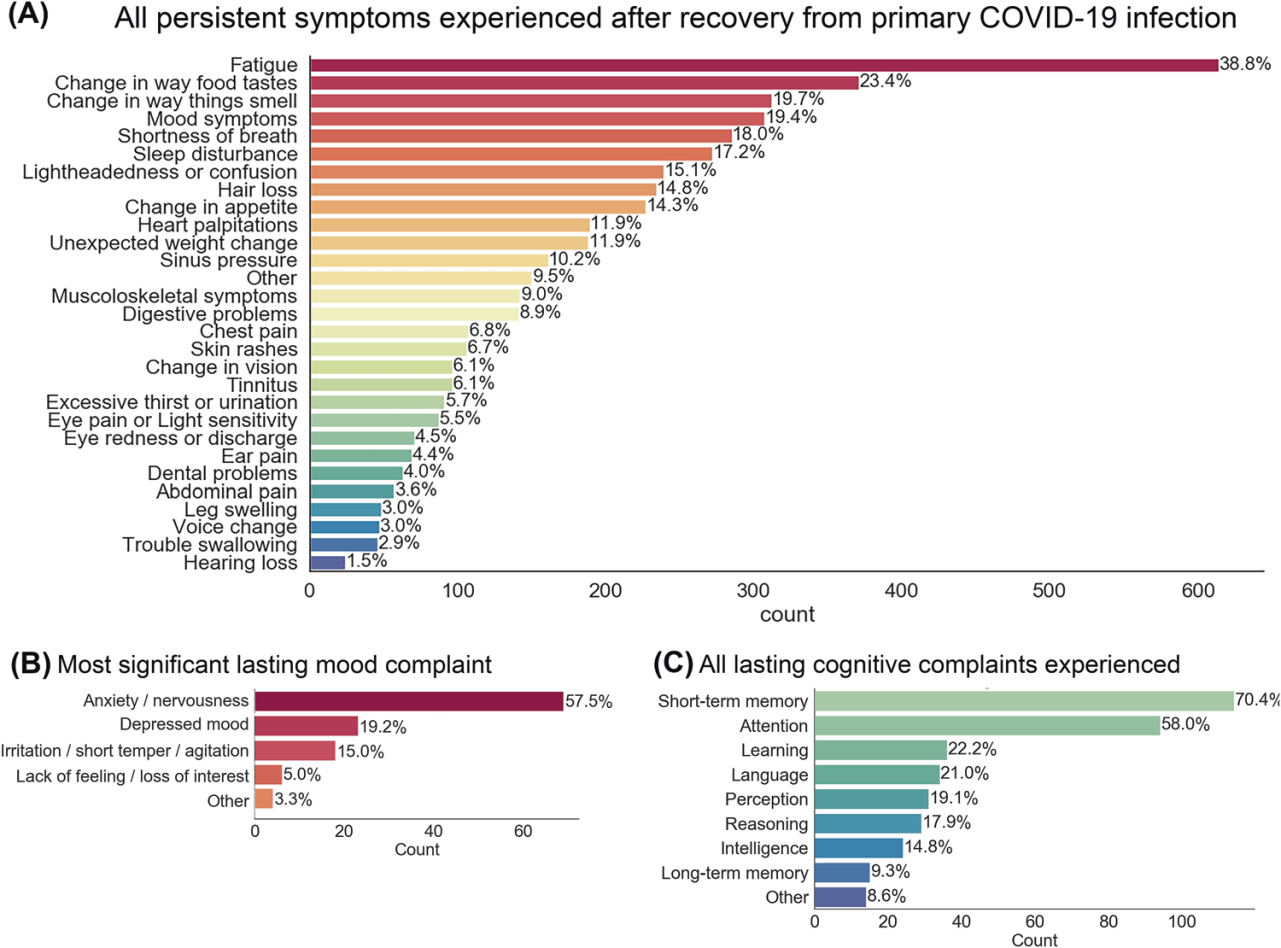
Prevalence of specific symptoms experienced by individuals reporting long-term sequelae following recovery from COVID-19. 70.6% of participants reported presence of one or more lasting symptom after recovery. The mean number of lingering symptoms reported was 3.06 (SD = 3.73). Chief lingering symptom complaints in the sample were fatigue, change in the perception of taste and smell, and mood symptoms (**A**). Follow up questions in a subset of participants provide insight into the primary kinds of mood (**B**) and cognitive (**C**) complaints expereinced. The proportion of participants that reported mood or cognitive/memory complaints following illness were 19.4% and 25.1%, respectively.

1,118 (70.6%) of participants reported presence of one or more lasting symptom after recovery from primary COVID-19 illness. Twenty-five percent of patients reported having cognitive or memory problems as a result of their COVID illness. Patients asked about kinds of cognitive complaints reported short-term memory (70%), attention (58%) and learning (22%) issues as the most frequently occurring. In addition, 19.4% of participants endorsed lasting mood symptoms. The most common lasting mood complaints, in those that endorsed lasting mood complaints, were anxiety/nervousness (58%), depressed mood (19%) and irritation/short temper/agitation (15%). Frequencies of all observed lasting effects are provided in Fig. 1. An overview of patients’ reported symptoms and secondary medical complications during COVID-19 illness is reported in Supplementary Material, Fig. S1*.*

### Early versus late timing of infection

**Fig. 2:**
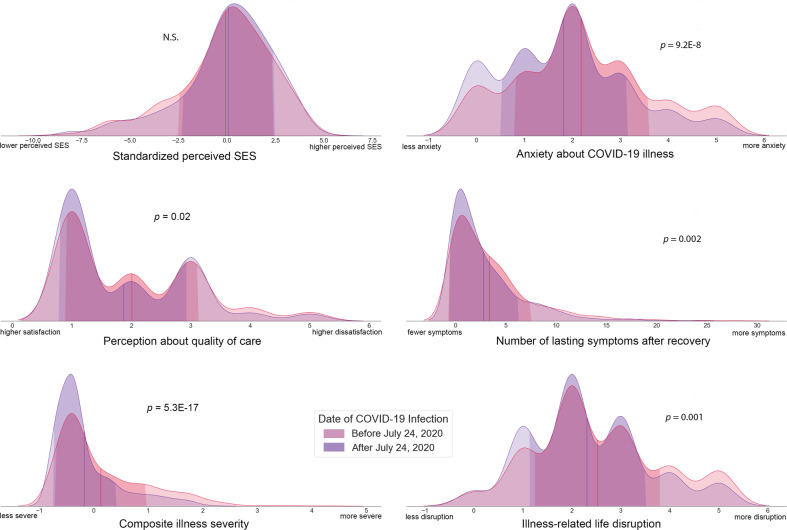
Differences in patients infected early versus late in the COVID-19 pandemic. Comparisons between patients infected with COVID-19 early versus late in the pandemic yield mixed results. While groups do not differ greatly in demographics, there are a few pronounced differences in clinical and psychosocial factors, most notably self-reported illness severity and anxiety about COVID-19 illness. Vertical red and purple lines on each distribution plot represent group means for early versus late infection, respectively. Standard deviations for each group are indicated by the darker shading on each plot.

Early versus late infection groups did not differ in sociodemographic variables, including age, education, gender, marital status, race, or objective SES. In addition, groups did not differ in prevalence of prior mental health treatment, pre-existing medical conditions, lasting neurological and/or cognitive symptoms after recovery, presence of ongoing lasting symptoms, perceptions about quality of care, perceived discrimination, perceived SES, or current stress. However, significant differences in early versus late infection groups were observed for composite illness severity (*p* = 5.3E-17), number of lasting symptoms (*p* = .002), and number of lasting mood complaints after recovery (*p* = .00008). Direction of these effects was such that patients in the early infection group reported more severe illness and more Long COVID/PASC symptoms. Early versus late groups also differed significantly in anxiety about illness (*p* = 9.2E-8) and ratings of COVID illness-related life disruption (*p* = .001), where the early infection group reported increased concerns of anxiety and life disruption. Analyses controlled for multiple comparisons (see Table 1). A subset of these comparisons are plotted for early and late infection groups in Fig. 2.

**Table 1:** Sociodemographic and illness characteristics in patients infected early or late in the pandemic.

	Early COVID (*n* = 774)	Late COVID (*n* = 810)	Statistical results
	M(SD) or N(%)	M(SD) or %	
*Sociodemographic*			
Age in years	44.42 (14.25)	44.85 (15.66)	*t*(1543) = −0.56, *p* = 0.58, *95CI*[−1.98, 0.99]
Education	6.90 (1.41)	6.78 (1.44)	*t*(1542)=1.70, *p* = 0.09, *95CI*[−0.01, 0.27]
Female gender	552 (71.4%)	563 (69.5%)	*X*^2^(3,1583)=0.92, *p* = 0.63
Partnered/married	254 (54.4%)	440 (54.3%)	*X*^2^(1,1584)=3.00, *p* = 0.70
Non-White race	267 (34.5%)	237 (29.3%)	*X*^2^(1,1584)=5.00, *p* = 0.03
Composite SES risk score	0.01 (2.36)	−0.01 (2.26)	*t*(1582)=0.08, *p* = 0.93, *95CI*[−0.22, 0.23]
*Clinical characteristics*			
Previous mental health treatment	190 (24.5%)	206 (25.4%)	X^2^(1,1584)=0.17, *p* = 0.69
Any pre-existing medical conditions	356 (46.0%)	374 (46.2%)	X^2^(1,1584)=0.01, *p* = 0.94
^a^ Composite illness severity	**0.13 (0.82)**	−**0.17 (0.58)**	***t*(1384)=8.49**, ***p*** = **5.3E-17**, ***95CI*[0.23, 0.37]**
^a^ Lasting symptoms after recovery *(range 0–29)*	**3.33 (4.00)**	**2.76 (3.42)**	***t*(1522)=3.10**, ***p*** = **0.002**, ***95CI*[0.22, 0.96]**
Lasting neurological changes after recovery *(range 0–6)*	0.44 (0.74)	0.43 (0.73)	*t*(1582)=0.29, *p* = 0.77, *95CI*[−0.06, 0.08]
Lasting cognitive/memory problems after recovery	85 (11.0%)	77 (9.5%)	X^2^(3,1584)=5.23, *p* = 0.16
^a^ Lasting mood complaints after recovery	**181 (23.4%)**	**126 (15.6%)**	**X**^**2**^**(1,1584)=15.23,** ***p*** = **0.00008**
Lasting symptoms ongoing	348 (45.0%)	367 (45.3%)	X^2^(1,1584)=0.02, *p* = 0.89
Were you satisfied with the medical care you received? *(1* = *very satisfied, 5* = *very dissatisfied)*	2.01 (1.12)	1.87 (1.07)	*t*(1465)=2.35, *p* = 0.02, *95CI*[0.02, 0.25]
*Psychosocial environment*			
Discrimination frequency	2.66 (1.41)	2.47 (1.41)	*t*(1398)=2.57, *p* = 0.01, *95CI*[0.05, 0.35]
Discrimination stress	2.12 (0.94)	2.03 (0.97)	*t*(1380)=1.79, *p* = 0.07, *95CI*[−0.01, 0.19]
Perceived SES	−0.08 (2.43)	0.07 (2.39)	*t*(1582) = −1.23, *p* = 0.22, *95CI*[−0.38, 0.10]
^a^ How anxious were you about being ill? (*0* = *no anxiety, 5* = *extreme anxiety)*	**2.18 (1.39)**	**1.82 (1.32)**	***t*(1582)=5.37,** ***p*** = **9.2E-8**, ***95CI*[0.23, 0.50]**
Please rate current stress level *(1* = *nothing, 7* = *extreme)*	4.07 (1.49)	3.93 (1.52)	*t*(1491)=1.77, *p* = 0.08, *95CI*[−0.02, 0.29]
^a^ How much did your COVID illness disrupt your life? *(0* = *no disruption, 5* = *extreme disruption)*	**2.52 (1.29)**	**2.31 (1.20)**	***t*(1560)=3.35,** ***p*** = **0.001**, ***95CI*[0.09, 0.34]**

Values in BOLD typeset are statistically significant after application of Holm–Bonferroni correction for multiple comparisons.

### Path model results

**Fig. 3:**
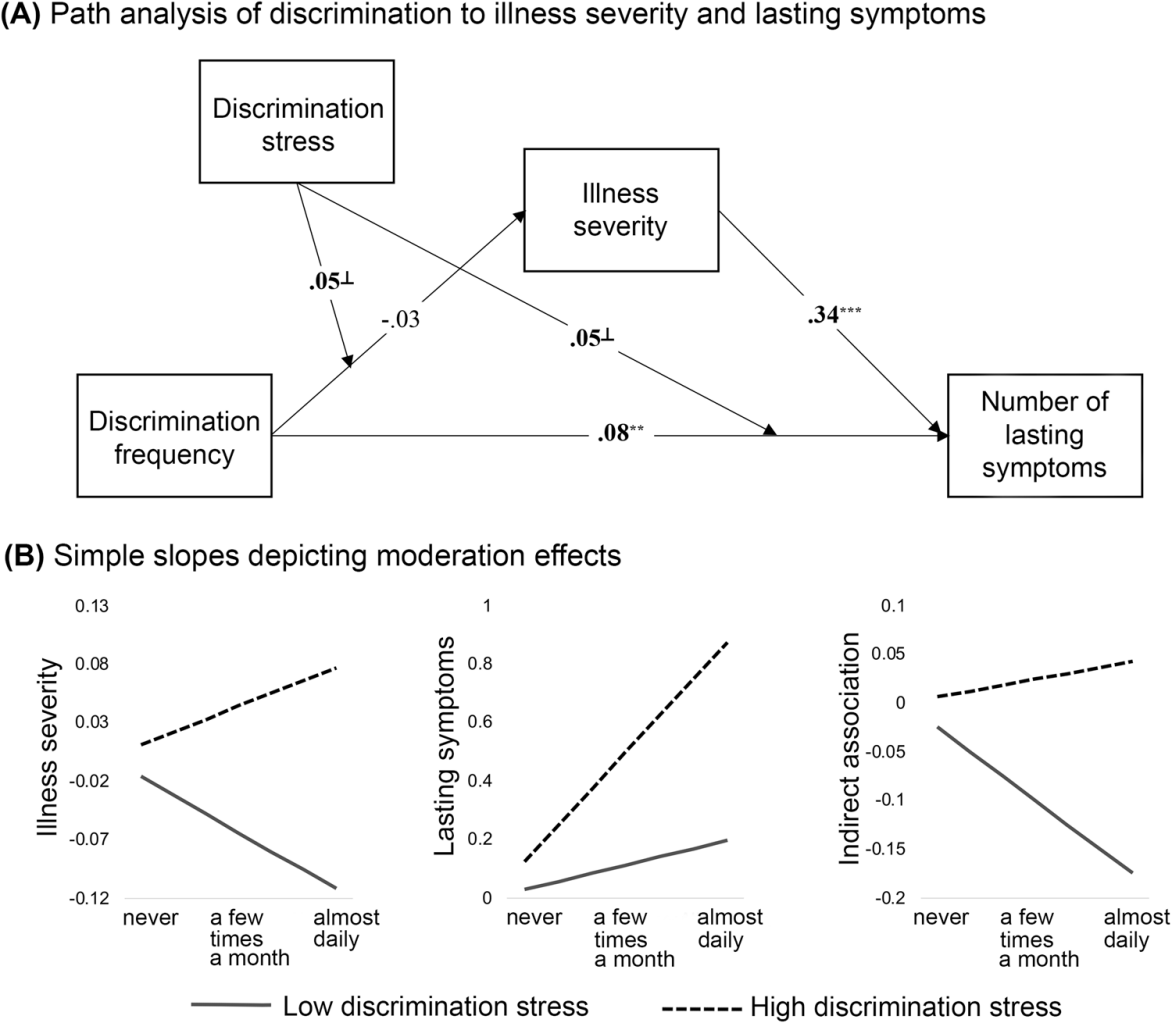
The observed path model and simple slopes depicting moderation effects. Observed associations between discrimination frequency, illness severity and number of lasting symptoms, with moderation by discrimination stress are represented in **A**. Standardized coefficients are shown. On all pathways, we controlled for race, cumulative SES risk score, perceived SES score, history of mood/anxiety disorder, history of diabetes/heart disease, COVID-illness life disruption, COVID-illness anxiety, and early versus late illness onset (i.e., peak 1 versus peak 2). A summary of observed moderation effects is provided in **B**, plotting model-estimated standardized simple slopes for all values of discrimination frequency. The *x*-axis for **B** is discrimination frequency. Discrimination stress moderates the direct effects of discrimination frequency on illness severity and lasting symptoms (left and middle plot). Discrimination stress also moderates the indirect effect of discrimination frequency on lasting symptoms through differential impacts on illness severity (right plot). ^⏊^*p* < .10, ^**^p < .01, ^***^p < .001.

Results indicated that two important aspects of discrimination experiences—the frequency at which they occur and the stress associated with these experiences—interact to predict illness severity and lasting symptoms. In addition, the interactive effects of discrimination frequency and stress on lasting symptoms was partially mediated through illness severity (*β* = .02, CI [.01, .03]). In a model that did not include moderation by discrimination stress, the indirect association through illness severity was *not* present (*β* = −.01, CI [−.03, .01]). This indicates that illness severity partially explains the link between discrimination and lasting symptoms, and highlights the importance of considering individual differences in subjective stress from discrimination. Full path model results are illustrated in Fig. 3A and reported in Supplementary Material, Table S3.

Simple slopes for individuals 1 SD + /- the mean for discrimination stress provide a visual representation of the direction of observed effects. Slopes indicate that increased frequency of discrimination was a stronger predictor of both increased illness severity and increased lasting symptoms for individuals reporting higher levels of stress from discrimination, see Fig. 3B. Further, there was a positive mediation effect of increased discrimination on increased lasting symptoms through greater illness severity for individuals reporting higher stress from discrimination (mean + 1 SD: *β* = .01, CI [−.01, .02]), but not for individuals reporting lower stress from discrimination (mean -1 SD: *β* = −.03, CI [−.05, −.003]); see Fig. 3B. These findings suggest that both the frequency of and stress associated with chronic discrimination contribute to disparities in COVID-19 health outcomes.

It is possible, however, that observed effects may not be specific to stress associated with chronic experiences of discrimination, but may instead be driven by poorer outcomes associated with increased stress levels more generally. As an analytical control, the same path analysis was tested using current stress levels as a moderator, instead of discrimination stress, which was included as an additional covariate. Results indicated that current stress moderated the impact of discrimination frequency on lasting symptoms (Supplementary Material, Fig. S2). However, current stress did not moderate the direct effect of discrimination frequency on illness severity, nor the indirect association between discrimination frequency and lasting symptoms through illness severity. These findings suggest that chronic stress and frequent experiences of discrimination have unique contributions to predicting disparities in Long COVID/PASC.

### Associations between discrimination and illness differ with individual perceptions of clinical care quality

A path analysis examined whether perceived quality of care influenced the observed conditional associations, as depicted in Fig. 3. Detailed description of this analysis is provided as Supplementary Material. In brief, participants were divided into high and low quality of care groups, based on self-ratings of care as excellent (*n* = 727) or less than excellent (*n* = 740). The majority of paths remained significant when looking within the low quality of care group; however, many of these paths were no longer significant when looking only within the high quality of care group (Supplementary Material, Fig. S3). This suggests that high quality of perceived clinical care may impact links between chronic discrimination and illness severity.

### Lasting neurological syndrome

**Fig. 4:**
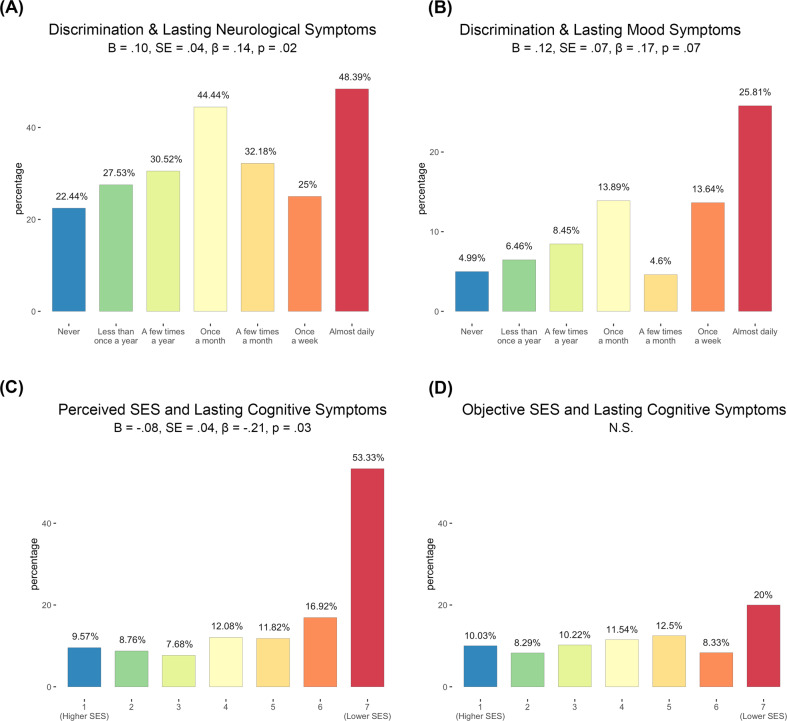
Perceived discrimination and perceived SES predict increased neurological and cognitive symptoms after recovery from COVID-19. *Post hoc* logistic regressions provide evidence that individuals reporting greater discrimination frequency had a significantly greater likelihood of reporting lasting neurological symptoms, *p* = .02 (**A**). Further, data demonstrate that perceived (**C**) but not objective (**D**) SES predicts lasting cognitive complaints after recovery from COVID-19 illness. There was a trend in the relationship between increased discrimination frequency and lasting mood symptoms, *p* = .07 (**B**). All analyses controlled for non-white race, cumulative SES risk score, perceived SES score, history of diabetes/heart disease, COVID-illness life disruption, COVID-illness anxiety, and early versus late illness onset (i.e., peak 1 versus peak 2). .

In final, exploratory analyses, we used logistic regression to address whether higher perceived discrimination was predictive of a general syndrome of lasting effects, or if there is evidence that *neural* domains are specifically affected. In these exploratory analyses, we also addressed whether perceived SES, COVID-life disruption, or illness-related anxiety predicted occurrence of lasting neural symptoms. We observed a significant positive effect of both discrimination frequency, Fig. 4A, and COVID-life disruption, *B* = 0.16, *SE* = 0.12, *β* = 0.19, *p* = 0.01 (not pictured), on number of lasting neurological symptoms. Discrimination frequency was not predictive of lasting cognitive symptoms, *B* = 0.05, *SE* = .06, *β* = 0.06, *p* = 0.47; however, perceived SES, Fig. 4C, and COVID-life disruption, *B* = 0.32, *SE* = 0.08, *β* = 0.40, *p* < 0.001 (not pictured), both had a significant effect on presence of lasting cognitive symptoms. Finally, a trend was observed in the association between discrimination frequency and lasting mood symptoms, Fig. 4B, and significant positive effects were observed between lasting mood symptoms and both illness anxiety, *B* = .34, *SE* = 0.09, *β* = .46, *p* < 0.001 and COVID-life disruption, *B* = 0.27, *SE* = .09, *β* = 0.33, *p* = 0.01 (not pictured).

## Discussion

Primary conclusions from this study are that: (1) lasting symptoms are common, with 70.6% of patients in this study reporting one or more symptoms, (2) specific psychosocial factors (perceived discrimination and perceived SES) place select individuals at greater risk of Long COVID/PASC, (3) mental health is an important aspect of Long COVID/PASC, (4) patient perceptions regarding quality of medical care can be important in interpreting these relationships, and (5) illness early in the pandemic is associated with more severe illness and more frequent lasting complaints. This study also confirms that illness severity was predictive of Long COVID/PASC. This may explain in part why in the current study of mildly (including asymptomatic) to severely infected patients, ranging in age from 18 to 96 years old, we observe slightly lower prevalence of Long COVID/PASC than has been reported elsewhere. The sample was representative of a wider health spectrum, and the study relied on direct patient report, distinctions that have been highlighted as methodological priorities in recent studies of Long COVID/PASC [2, 22]. While not central to the present study, we also find that age and sex of patients also relate to prevalence of Long COVID/PASC (Supplemental Material).

A major focus of this project was to evaluate individual determinants of Long COVID/PASC, with specific attention on experiences of discrimination as a predictor of health outcomes. Data presented suggest that chronic discrimination is a significant predictor of lasting COVID-19 sequalae through both direct and indirect pathways, in models that account for mental and physical health comorbidities and sociodemographic factors. The addition of discrimination stress, but not current stress, to path models affects the association between chronic discrimination and illness severity, highlighting specificity of observed effects. Thus, it is not a general syndrome of increased psychosocial burden; instead, it appears that frequent experiences of general discrimination place individuals at greater risk for becoming more ill when infected, and at greater risk for experiencing increased lasting health complaints after recovery.

An important question brought to light in the current study is how perceived discrimination relates to structural inequalities in the lives of individuals afflicted with Long COVID/PASC. This study cannot directly address that question. However, we found that individuals reporting lower perceived socioeconomic status were significantly more likely to also report lower care quality (OR = 0.92, 95% CI [.87, .97]). It is possible that low quality care perceptions may reflect structural inequalities in access to and/or the quality of healthcare received [23]. A notable takeaway from the present study is the importance of subjective versus objective perspectives. This is illustrated well in the finding that perceived SES was a more robust predictor of long-term outcomes than actual SES, both of which were composite factors with high internal validity. Another notable observation from the present study was that associations between experiences of discrimination and illness differed based on the patient’s perceived quality of medical care. This is an encouraging avenue for promoting health in individuals at enhanced risk.

Overall, this study highlights the urgency for research to rigorously address long-term physical and neurological outcomes of COVID-19, as a large proportion of our global population has now been infected. Knowledge about the primary compromised domains informs our approach to treating afflicted individuals. Future studies would benefit from collecting information about patient perceptions and experiences, as these are clearly significant drivers of Long COVID/PASC health outcomes. Our data support the position that considering the individual is vital. A current crisis in care for individuals with Long COVID/PASC is the occurrence of “medical gaslighting”, referring to a practice of discounting or dismissing patient beliefs about their medical conditions. The present study prioritizes patient account and combines metrics (e.g., fever severity, illness length, medical complications, and COVID-related hospitalization) and perceptions (e.g., illness severity) to derive a more comprehensive, potentially more sensitive, composite factor (Supplemental Material, page 3). Knowledge about predictors and prevalence of lasting illness sequelae makes it possible to make informed economic and policy decisions about research and treatment.

## Materials and methods

### Study design

A search of the NYU Langone Health record system in February 2021 identified 23,267 individuals ages 18 and older with COVID-19 diagnosis based on EPIC International Classification of Diseases (ICD-10) code U07.* Of those on this list, individuals (1) with email contact, (2) not deceased, and (3) not designated as having previously opted out of research contact were eligible to participate. After application of these exclusions, 17,282 individuals were sent an email inviting them to participate in a 15-min survey. Compensation was entry into a drawing for a $25 gift card. All surveys were completed between February 23, 2021 and April 4, 2021. Description of the survey measures and additional details on survey administration are described in Supplemental Material.

### Participants

**Fig. 5:**
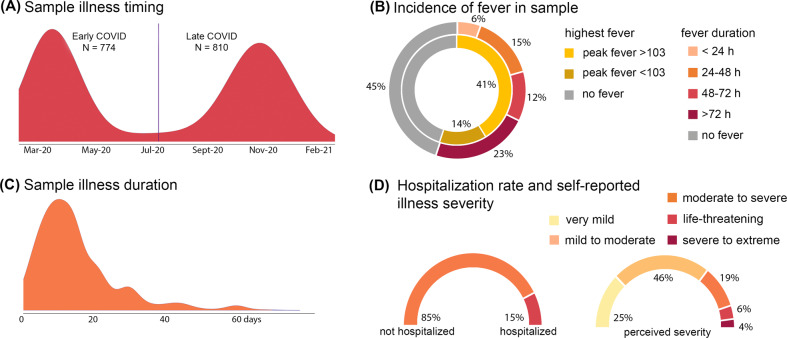
Overview of illness severity in current sample of *N* = 1584 adults infected with COVID-19. Quality validated data were used to generate descriptive statistics that summarize illness timing (**A**); frequency and extent of fever (**B**); duration of illness (**C**); and both rate of hospitalization and self-reported illness severity in our adult sample (**D**). Bimodal distribution in **A** aligns well with observed incidence in New York City over this time period. The vertical line in **A** is the mean cut point used to analyze potential differences in early versus late infection groups.

A total of 2,212 individual responses to the survey were received. 1,584 cases were retained after data validation measures were applied (Supplemental Material). The minimum sample size selected for this study was 400, with focus on tests of mediation including eight covariates. For demographic characteristics of the final sample after quality assurance steps, see Supplemental Material, Table S1. For overview of sample illness timing and severity, see Fig. 5. All study procedures were approved by the New York University Grossman School of Medicine Institutional Review Board and consent was obtained from all participants. The approved study protocol included sharing of de-identified data with outside researchers or research databases.

### Socioeconomic status

A measure of cumulative *objective* socioeconomic status (SES) was generated by standardizing and summing the following demographic variables: household income-to-needs ratio (i.e., income relative to household size), education level, stability of housing, and receipt of public assistance. Next, a measure of *perceived* SES was computed by standardizing and summing financial satisfaction, financial worries, perceived financial stability, and the MacArthur ladder of perceived social standing. Confirmatory factor analyses were used to verify fit of SES composite variables, which indicated excellent fit for both variables (objective SES: *RMSEA* = 0.029, *CFI* = .985, *χ2* = 177.53, *p* < 0.001; perceived SES: *RMSEA* = 0.0, *χ2* = 632.03, *p* < 0.001). Demographic questions and response options used to derive these measures are available at: https://osf.io/82rkj, via the NCIPR Demographic Survey.

### Statistical approach

A consideration is whether individuals that were ill during the first incidence peak of COVID-19 differ from individuals ill during the second peak in our sample. The peaks in NYC occurred on April 8, 2020 and on Jan 7, 2021. (https://www1.nyc.gov/site/doh/covid/covid-19-data-trends.page) The sample was mean split based on self-reported date of illness, resulting in split at July 24, 2020 for early versus late infection. Early and late cases were compared on sociodemographic, clinical, and psychosocial factors, using chi-squared tests and two-sample *t*-tests run with 5000 bootstrapped samples. Each outcome was be assessed for adherence to a normal distribution.

Path analyses were used to test (1) direct associations between lifetime discrimination history, self-reported illness severity, and lasting symptom count, (2) indirect associations between discrimination and lasting symptoms, mediated through illness severity, and (3) moderation by stress from experiencing discrimination. Secondary analyses tested (1) specificity to discrimination stress, relative to experiencing increased stress in general, and (2) differences based on subjective perceptions of medical care (excellent versus non-excellent reported quality). See Supplemental Material for detailed descriptions of all measures.

Full information maximum likelihood (FIML) was used to avoid biased estimates associated with listwise deletion. Tests of statistical mediation were conducted using 5000 bootstrap samples to generate bias-corrected confidence intervals. Race (white vs. non-white), objective SES risk score, perceived SES score, history of mood/anxiety disorder, history of diabetes or heart disease, COVID-illness life disruption, COVID-illness anxiety, and early versus late illness onset were controlled for in all analyses. All path analyses were conducted using Mplus v8.

## Supplementary information

Supplemental Material

## Data Availability

All data have been made publicly available and curation/validation processes have been documented. Please refer to https://pubmed.ncbi.nlm.nih.gov/34100021/ for documentation about validation/curation and refer to https://osf.io/82rkj/wiki/home/ for data; License: CC-By Attribution 4.0 International.
